# Antioxidant Effect of Standardized Extract of Propolis (EPP-AF®) in Healthy Volunteers: A “Before and After” Clinical Study

**DOI:** 10.1155/2020/7538232

**Published:** 2020-10-16

**Authors:** Débora P. Diniz, Daniela Aparecida Lorencini, Andresa Aparecida Berretta, Monica A. C. T. Cintra, Erica N. Lia, Alceu A. Jordão, Eduardo B. Coelho

**Affiliations:** ^1^Department of Internal Medicine, Ribeirão Preto Medical School, Ribeirão Preto, Brazil; ^2^Laboratory of Research, Development and Innovation, Apis Flora Indl. Coml. Ltda., Ribeirão Preto, São Paulo, Brazil; ^3^General Clinical Research Center, Teaching Hospital Ribeirão Preto, Ribeirão Preto, Brazil; ^4^Department of Dentistry, Faculty of Health Sciences, University of Brasília, Brasilia, Brazil; ^5^Department of Health Sciences, University of São Paulo, Ribeirão Preto, Brazil

## Abstract

**Background:**

Propolis is rich in polyphenols, especially flavonoids and phenolic acids, and has significant antioxidant activity, shown mainly in “*in vitro*” studies.

**Objective:**

The aim of this study was to evaluate the antioxidant efficacy and safety of a standardized propolis extract in healthy volunteers.

**Design:**

A two-phase sequential, open-label, nonrandomized, before and after clinical trial.

**Methods:**

Healthy participants received two EPP-AF® doses (375 and 750 mg/d, P.O, tid) during 7 ± 2 days, starting with the lower doses. Immediately before starting EPP-AF® administration and at the end of each 7-day dosing schedule, blood and urine samples were collected for quantification of 8-OHDG (8-hydroxydeoxyguanosine) and 8-ISO (8-isoprostanes) in urine and GSH (reduced glutathione), GSSG (oxidized glutathione), SOD (superoxide dismutase), FRAP (Ferric Reducing Antioxidant Power), vitamin E, and MDA (malondialdehyde) in plasma.

**Results:**

In our study, we had 34 healthy participants (67.7% women, 30 ± 8 years old, 97% white). The 8-ISO, a biomarker of lipid peroxidation, decreased with both doses of EPP-AF® compared to baseline (8-ISO, 1.1 (0.9–1.3) versus 0.85 (0.75–0.95) and 0.89 (0.74–1.0), ng/mg creatinine, *P* < 0.05, for 375 and 750 mg/d EPP-AF® doses versus baseline, mean and CI 95%, respectively). 8-OHDG, a biomarker of DNA oxidation, was also reduced compared to baseline with 750 mg/d doses (8-OHDG, 15.7 (13.2–18.1) versus 11.6 (10.2–13.0), baseline versus 750 mg/d, respectively, ng/mg creatinine, *P* < 0.05). Reduction of biomarkers of oxidative stress damage was accompanied by increased plasma SOD activity (68.8 (66.1–73.3) versus 78.2 (72.2–80.5) and 77.7 (74.1–82.6), %inhibition, *P* < 0.0001, 375 and 750 mg/d versus baseline, median and interquartile range 25–75%, respectively) and by increased GSH for 375 mg/d EPP-AF® doses (1.23 (1.06–1.34) versus 1.33 (1.06–1.47), *μ*mol/L, *P* < 0.05).

**Conclusion:**

EPP-AF® reduced biomarkers of oxidative stress cell damage in healthy humans, with increased antioxidant enzymatic capacity, especially of SOD. This trial is registered with the Brazilian Registry of Clinical Trials (ReBEC, RBR-9zmfs9).

## 1. Introduction

Maintaining cell viability requires a continuous process of energy production, which is largely accomplished by mitochondria. In this process, reactive oxygen species are formed, many of which participate in the control of cellular homeostasis, acting as biochemical intracellular signaling or even controlling gene expression. Under conditions of hypoxia, aging, or cell damage, this respiratory chain may be decoupled, leading to the formation of reactive substances such as aldehydes, malondialdehyde (MDA), superoxides, and peroxides, which in turn damage nucleic acids (DNA/RNA) and cellular membrane [1]. Under physiological conditions, there is a balance between free radical production and its elimination. However, excess production of oxygen free radicals, defined as oxidative stress, can lead to cell damage and be part of a number of some chronic degenerative diseases observed in humans, such as cancer, inflammatory and cardiovascular diseases, diabetes mellitus, atherosclerosis, and Alzheimer's disease [2–4].

Antioxidant defenses are composed of enzymatic mechanisms such as glutathione peroxidase (GPx), catalase, and superoxide dismutase (SOD) [5] and dozens of substances that play a role in binding and neutralizing free radicals. These substances may be endogenous (e.g., uric acid) or offered from diet or supplements (flavonoids, carotenoids, polyphenols, ascorbic acid, and vitamin E) [6–8]. In complementary medicine, the use of natural antioxidant-rich substances is frequent, mostly polyphenols [9, 10]. Among the most abundant sources of polyphenols, especially flavonoids and phenolic acids, are propolis and wine [11].

Propolis is a complex phytocompound made from resinous and balsamic material harvested by bees from flowers, branches, pollen, and tree exudates. The chemical composition of propolis, besides flavonoids, also contains aromatic acids and esters, aldehydes and ketones, terpenoids and phenylpropanoids, steroids, amino acids, polysaccharides, hydrocarbons, fatty acids, and inorganic components such as iron, calcium, manganese, and aluminum [12]. However, the composition of propolis is very variable. It depends on the geographical region and the plants from which the bees extract its components. In Brazil, for example, there are 13 different types of propolis, including green, the most widely used, red, and brown, whose main sources are *Baccharis dracunculifolia*, *Dalbergia ecastaphyllum,* and *Hyptis divaricata*, respectively [13].

The antioxidant activity of the green propolis is correlated with the chemical composition of its different fractions, especially flavonoids and p-coumaric acid derivatives [14]. Most studies related to the antioxidant properties of propolis were performed in cell culture or in animals. In the available literature, there are only a few studies investigating the antioxidant effect of propolis in humans [13]. The available information about clinical trials is even more limited.

The first clinical trial using oral propolis for more than 30 days showed reduced lipid peroxidation, measured by plasma MDA concentration and increased SOD activity [11]. However, this study showed an antioxidant effect in men, but not in women. In another clinical trial, the oral use of a commercial propolis solution for 90 days showed reduced lipid peroxidation and increased concentration of reduced glutathione (GSH) compared with baseline. In addition, there was also an increase in HDL concentration [15]. In a study with type 2 diabetes mellitus (DM2) patients, the use of propolis increased the plasma concentration of GSH and reduced oxidation markers [16, 17]. However, these results were not observed in another study performed with Brazilian green propolis in diabetic patients [18].

The standardized Extract of Propolis (EPP-AF®) is obtained by microencapsulation technology and has a chemical profile and stable batch by batch, consisting mainly of caffeic acid, p-coumaric acid, cinnamic, aromadendrin, isosakuranetin, and artepillin C [19], substances with important antioxidant effect *in vitro* [20, 21]. On the other hand, the bioavailability of polyphenols *in vivo* may be low, as some compounds have low absorption by the gastrointestinal tract or have extensive metabolism. For this reason, microencapsulation was used.

Considering that some chronic diseases have pathophysiological mechanisms involving oxidative damage by free radical generation, there is an interest in studying the use of “functional food” or “nutritional supplements” with antioxidant properties to prevent the worsening of such chronic diseases. Thus, the main objective of this study was to evaluate the antioxidant effect of standardized propolis extract (EPP-AF®) for oral use in healthy volunteers.

## 2. Methods

### 2.1. Study Design

The study design was a two-phase sequential, open-label, nonrandomized, before and after clinical trial.

#### 2.1.1. Investigational Product

Coated EPP-AF® tablets (batch 190000116) were obtained from Apis Flora Indl. Coml. Ltda (Ribeirão Preto, SP/Brazil) using Propolis Standardized Extract EPP-AF® batch *n*. 189900116 [21]. EPP-AF® tablets offered 12 mg/tablet of Artepillin C by HPLC method [22], 7.10 mg/tablet of total flavonoid as quercetin, and 19.49 mg/tablet of gallic acid.

#### 2.1.2. Primary, Secondary, and Exploratory Outcomes

The primary outcome was to evaluate the antioxidant efficacy of EPP-AF, using the urinary excretion of 8-isoprostanes, a marker of *in vivo* lipid peroxidation, and 8OHDG, a marker of oxidative DNA damage as surrogate biomarkers. The secondary outcome was the safety of EPP-AF® in healthy volunteers, assessed by kidney and liver biochemical tests. The exploratory outcomes were focused on studying some biochemical pathways involved in oxidative stress. We studied the Ferric Reducing Antioxidant Power (FRAP), Vitamin E, superoxide dismutase (SOD) activity, and GSSG/GSH in plasma. Also, the MDA assay was done to detect lipid peroxidation in plasma.

### 2.2. Participants

Participants from both sexes were included, they were between 18 and 60 years old, and their weight varies, 15% of what is considered normal for women and men, using body mass index (BMI). The recruitment period started on January 18^th^, 2016, and the follow-up period ended on December 1^st^, 2016.

The inclusion criteria were healthy participants, no history of allergies, no continuous use of medication one week prior to the study, and having read and signed the informed consent form (ICF).

The exclusion criteria were pregnant women, women breastfeeding, smokers, and participants with a history of alcohol or drug abuse.

Participants underwent physical examination, electrocardiogram, and laboratory tests for renal function assessment (creatinine and urea), liver function assessment (transaminases and bilirubins), metabolic profile (fasting blood glucose and lipidogram), electrolytes and serologies for hepatitis B and C, and HIV. All biochemical tests were done in the automatized biochemistry analyzer (Weiner, Rosario, Argentina) of Teaching University Hospital, following the standardized methods according to the manufacturer. Also, hematologic, metabolic, renal, and liver function tests were done again 30 days before the end of the use of EPP-AF® (750 mg/d), to assess safety.

The study was approved by the Human Research Ethics Committee of the Clinical Hospital at Ribeirão Preto Medical School-University of São Paulo (HCFMRP-USP) (No. 5912/2012) and conducted at the Clinical Research Unit (UPC) of HCFMRP-USP. The study was registered in the Brazilian Registry of Clinical Trials (ReBEC, RBR-9zmfs9).

### 2.3. Research Protocol

After signing the ICF and collecting and confirming the normal results in the clinical and biochemical assessment, the selected participants were invited to attend the UPC and fast for 12 hours. Blood and urine samples for oxidative stress markers were collected immediately before the oral administration of EPP-AF®. These values were considered as baseline. Afterward, the participants were instructed to use 375 mg EPP-AF® per day, divided into 3 oral administrations for a period of 7 ± 2 days. At the end of this period, participants returned to UPC to repeat the abovementioned blood and urine collection, clinical assessment, interrogation of adverse events, and adherence to the treatment by counting the number of capsules taken during this period. Participants who took less than 80% of the intended propolis tablets were excluded from the study. Next, participants were instructed to use the EPP-AF® dose in 3 daily oral administrations for a period of 7 ± 2 days. Finally, they returned to the UPC for the same procedures used for the previous dose. To assess safety, the hematologic, metabolic, renal, and liver function biochemical tests were done 30 days after the last dose used of EPP-AF® and the study was closed. Blood and urine samples were centrifuged and stored at −80°C until study tests were performed.

### 2.4. Evaluation of Antioxidant Proteins and Enzymatic Activity

GSH, GSSG (oxidized glutathione), and SOD (superoxide dismutase), and reduced and oxidized glutathione were determined in plasma according to Rahman et al. [23]. The assay is based on the reaction of GSH with 5,5′-dithio-bis (2-nitrobenzoic acid) (DTNB) which produces 5′-thio-2-nitrobenzoic acid (TNB), which has a maximum absorbance at 412 nm and oxidized glutathione-TNB adduct (GS-TNB).

Plasma samples (100 *μ*L) were placed in 5% metaphosphoric acid, centrifuged at 3000 g at 4°C for 10 min, and the upper clear layer was maintained at 0–4°C during the assay. 20 *μ*L of the blank or standard sample was inserted into the reaction plate. The absorbance was measured at 412 nm in a microplate reader (SpectraMax M5, Molecular Devices, USA). For the GSSG assay, 2 *μ*L of 2-vinylpyridine was added to the formation of 100 *μ*L of 2-nitro-5-thiobenzoic acid in the plasma, and the reaction was left for 2 hours in the dark. Then, 20 *μ*L was used for the plate reaction as described above. GSSG was calculated by the rate of formation of 2-nitro-5-thiobenzoic acid compared to the standard curve of GSSG. The reduced GSH was calculated as the total values of glutathione—GSSG. For SOD determination, a commercial kit (Sigma-Aldrich, USA cat. No. 19160) was used according to the manufacturer's instructions and read at 450 nm microplate reader (SpectraMax M5, Molecular Devices, USA).

#### 2.4.1. FRAP (Ferric Reducing Antioxidant Power) Quantification in Plasma

The FRAP reagent was made by mixing the acetate buffer solution and the 10 mM TPTZ solution (2,4,6-tripyridyl-s-triazine, Sigma, cat no. T1253-5 G) with ferric chloride solution in 10 : 1 : 1 solution. 10 *μ*L of plasma was pipetted and 300 *μ*L of FRAP solution was added. The plate was incubated on the spectrophotometer at 37°C for 4 minutes with a reading at 593 nm. Results were expressed as mM Fe^2+^.

#### 2.4.2. Dosage of Vitamin E in Plasma

Vitamin E dosage was performed by high-pressure liquid chromatography (HPLC), according to Arnaud et al. [24]. 200 *μ*L of plasma was pipetted into a test tube and 400 *μ*L of hexane was added and stirred. In sequence, 400 *μ*L of heptane was placed. It was stirred for 1 min and centrifuged at 3000 rpm/10 min. Then, a 200 *μ*L aliquot of the organic phase was taken, which was dried in N_2_ flow (g) and suspended in 200 *μ*L of the mobile phase. After the sample was prepared, it was injected with 20 *μ*L on HPLC. For the analyses, a Shimadzu (Japan) model LC-20AT chromatograph was used: column type C-18 (250 × 4.6 mm–5 *μ*m), UV-visible detector model SPD-20A, mobile phase composed of acetonitrile: dichloromethane: methanol 7 : 2 : 1, flow rate 1.0 mL/min, and detection at 292 nm. Concentrations were determined using external standards, and results were expressed in *μ*mol/L serum/plasma.

#### 2.4.3. Plasma MDA Quantification

MDA formation was quantified by the plasma colorimetric method. This analysis was performed according to the method proposed by Gerard-Monnier et al. [25], with some adaptations. 100 *μ*L of plasma was added in 300 *μ*L solution of 10 mM of 1-methylphenylindole in acetonitrile and methanol (2 : 1, v/v) and 75 *μ*L of pure HCL (37%). Soon after, the Eppendorf was vortexed and incubated in a water bath at 45°C for 40 minutes. After the bath, the samples were cooled on ice and then the Eppendorf was centrifuged at 4000 rpm for 10 minutes. From the supernatant, a 586 nm wavelength absorbance reading was performed (SpectraMax M5, Molecular Devices, USA). MDA concentration was calculated by comparing it to a hydrolyzed 1,1,3,3-tetramethoxypropane (TMP) curve.

#### 2.4.4. 8-Hydroxy-Deoxyguanosine (8-OHDG) and 8-Isoprostane in Urine (8-ISO)

Urine samples were centrifuged at 3500 g for 5 minutes at 4°C. They were then diluted in Milli-Q water (1 : 50 to 1 : 250) and pipetted onto ELISA plates for 8-OHDG or 8-isoprostane according to the manufacturer's instructions (Cayman Chemical, USA). Readings were taken on a microplate reader (SpectraMax M5, Molecular Devices, USA), and the dosages obtained were corrected by urinary creatinine dosage performed in the clinical analysis laboratory of the University Hospital by automated method (Weiner, Argentina).

### 2.5. Sample Calculation

For sample size estimation, GPower 3.0 Software (Dusseldorf, Germany) was used. Urinary distribution data for 8-isoprostanes were based on Villa et al. [26] and for 8-OHDG on Wu et al. [27]. For both outcomes, a sample of 34 participants demonstrated the power of 0.80 to detect differences of at least 45% of baseline with *P* value <0.05.

### 2.6. Data Analysis

Data were described as mean or median, with respective standard deviation or interquartile variation. Data considered as normal distribution were analyzed by repeated-measures ANOVA followed by Dunnet's post hoc test. Nonparametric distribution data were analyzed by ANOVA for nonparametric repeated measures (Friedman) followed by Dunn's post hoc test. The correlation between plasma SOD and GSH concentrations with 8-ISO or 8-OHDG oxidative damage markers was made by Spearman's test. It was considered as statistically significant, *P* value <0.05. Data were analyzed using GraphPad Prism Software version 7.0 (Graphpad Software, San Diego, CA, USA).

## 3. Results

**Figure 1:**
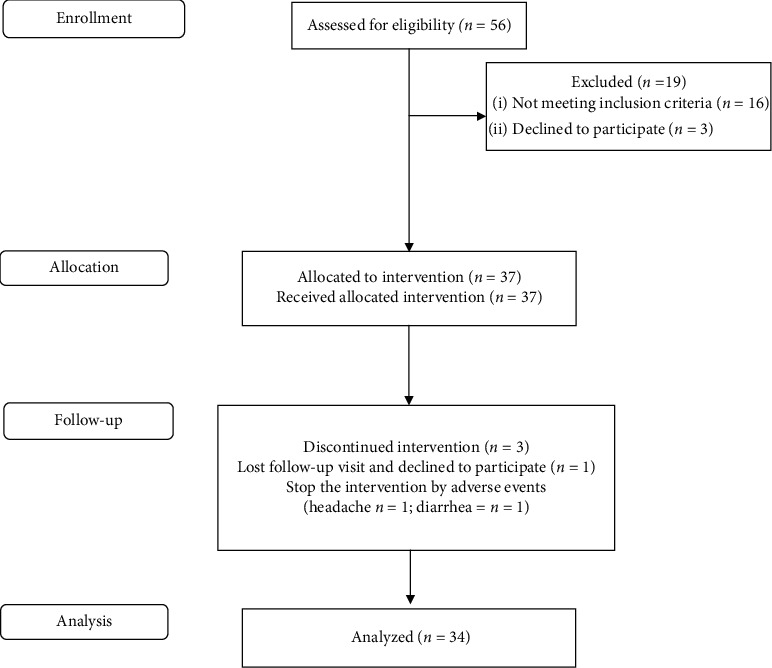
CONSORT flow diagram showing the progress of patients throughout the trial.

Figure 1 shows the participants who were recruited and completed the research protocol. In total, thirty-seven participants were allocated in the study, and 3 were excluded during the follow-up phase. One participant decided to withdraw the study protocol and two stopped EPP-AF® in the first week of use due to adverse events (headache and diarrhea). Both adverse events were limited, considered nonserious by the investigator, and potentially unrelated to EPP-AF®. The remaining 34 participants completed the study and had their data analyzed. The mean age of participants was 30 ± 8 years, 67.7% were women, and 97% self-reported as white. The biochemical tests used to assess the safety of EPP-AF® are detailed in Table 1. No changes in biochemical test results were noted comparing baseline and the end of study samples.

### 3.1. Nonenzymatic Systems

FRAP values (mmol/L) showed no differences after the use of 375 mg/d or 750 mg/d EPP-AF®. Similarly, there was no effect of EPP-AF® on plasma vitamin E concentration. The values are described in Table 2.

### 3.2. Antioxidant Proteins and Enzymatic Systems

**Figure 2:**
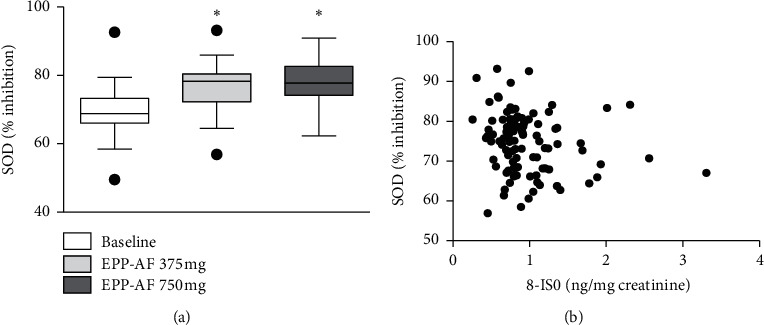
(a) Tukey's box-plot showing the plasma SOD activity (% inhibition) before (baseline) and after the use of EP-AF® 375 and 750 mg (^*∗*^*P* < 0.0001; 375 and 750 mg versus baseline). (b) Correlation between the plasma SOD activity (% inhibition) and the urinary excretion of 8-isoprostane (ng/mg creatinine). Each point represents a sample of urine and plasma collected in the baseline and after 375 and 750 mg/d treatment periods. R Spearman = − 0.21; *P*=0.03.

The antioxidant proteins and enzymatic systems studied (GSH, GSSG, GSH/GSSG ratio, and SOD) are described in Table 2. There was a significant elevation in plasma GSH concentration at the 375 mg/d EPP-AF® compared with baseline (Figure 2). SOD activity also increased compared to baseline with the use of EPP-AF® 375 mg/d and 750 mg/d. There were no significant changes in the amounts of GSSG in the periods before and after the use of EPP-AF®, as well as in the GSH/GSSG ratio. SOD activity was expressed as a percentage of inhibition and increased significantly with the use of EPP-AF® from baseline. The maximum effect observed was with a dose of 375 mg/d. The elevation from the mean baseline was 13.6% for the 375 mg/d and 12.9% for the 750 mg/d EPP-AF® (Figure 2(a)). SOD activity was correlated with urine 8-ISO values (R Spearman −0.20, *P*=0.03, Figure 2(b)), but not with 8-OHDG. There was no statistically significant correlation between GSH concentration and oxidative stress damage markers.

### 3.3. MDA, 8-ISO, and 8-OHDG

**Figure 3:**
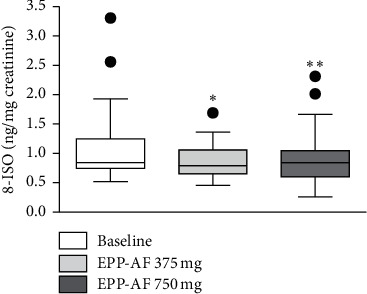
Tukey's box-plot showing the urinary excretion of 8-isoprostane (ng/mg creatinine) before (baseline) and after the use of EPP-AF® 375 and 750 mg (^*∗*^*P*=0.01 and ^*∗∗*^*P*=0.03, compared with baseline).

**Figure 4:**
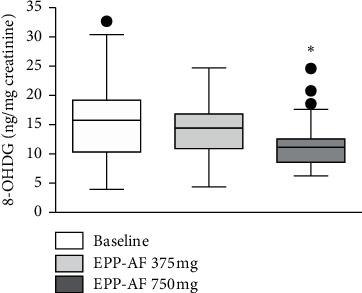
Tukey's box-plot showing the urinary excretion of 8-OHDG (ng/mg creatinine) before (baseline) and after the use of EPP-AF® 375 and 750 mg (^*∗*^*P*=0.004, compared with baseline).

There was a significant reduction in urinary excretion of 8-ISO with the use of EPP-AF®. Apparently, the maximum effect was observed at the dose of 375 mg/d, since increasing the dose was not accompanied by an increase in the magnitude of the effect. The difference from baseline means concentration was −0.25 (−0.05 to −0.44) with EPP-AF® at a dosage of 375 mg/d and −0.21 (−0.01 to −0.40) with EFF-AF® 750 mg/d (Figure 3). There was a reduction in urinary excretion of 8-OHDG only at the 750 mg/d EPP-AF® dose (Figure 4). There was no change in plasma MDA values (Table 3).

## 4. Discussion

The main finding of this study, conducted in healthy participants, was a reduction in biomarkers associated with cellular membrane and DNA damage caused by free radicals, using the microencapsulated Standardized Extract of Brazilian green Propolis (EPP-AF®). This reduction in damage was associated with an increase in GSH concentration and, more evident, with an increase in SOD plasmatic activity. The antioxidant effect of propolis is well known, mainly from *in vitro* studies in human cells. This activity is attributed mainly to the presence of flavonoids and polyphenols [28], among them, some caffeic and cinnamic acid derivatives [29–31]. EPP-AF® presents, unlike other propolis presentations studied, high content of artepillin C, another agent with marked antioxidant activity [32]. In this study, we showed the *in vivo* antioxidant effect of EPP-AF® on healthy young participants where low oxidation formation was expected and oxidative balance was preserved. However, the MDA formation in plasma was not changed with EPP-AF use. This apparent contradiction could be explained by a lack of sensitivity of the “*in vitro*” MDA method, compared with the measurement of 8-isoprostanes, which are chemically stable markers and are formed “*in vivo*.” They are specific to lipid peroxidation and are not affected by the dietary lipid content, in contrast with MDA [33]. The antioxidant effects of propolis observed in this work are in line with other studies in humans with associated diseases, particularly T2DM, where increased GSH and SOD concentrations were observed [16, 17], and in patients with dyslipidemia and cardiovascular risk, where an increase in HDL was observed [15]. Propolis use has been associated with improved health conditions in which inflammation is a common denominator. In addition to the abovementioned T2DM, there was an improvement in the cognitive pattern in patients with Alzheimer's disease, associated with a reduction in inflammatory markers [4]. Clinical trials have shown the efficacy of propolis in the treatment of oral diseases associated with herpesvirus type I [34] and others, such as prosthetic stomatitis [35], recurrent cold sore [36], and periodontal disease, and the reduction of oxidative stress was shown in this last study [37]. Regarding EPP-AF®, a clinical trial with chronic renal patients with proteinuria showed a reduction in monocyte chemoattractant protein-1 (MCP-1), which is a marker of inflammation and proteinuria intensity [38]. Thus, the present study suggests that part of the anti-inflammatory mechanisms attributed to propolis may be associated with its antioxidant potential. Although this study was not intended to elucidate the potential anti-inflammatory effect of EPP-AF®, inflammation and oxidative stress are interlinked processes, and metabolic and cardiovascular diseases have a low-intensity chronic inflammation state that may be related to the advancement of vascular disease and atherosclerosis processes [39, 40]. Oxidative stress activates the inflammatory cascade via NF-kB (nuclear factor kappa light chain enhancer of activated B cells), and interestingly, this pathway is inhibited by SOD overexpression [41], which in our study showed a correlation with urinary 8-isoprostane excretion.

Most of the antioxidant effects were observed at a dose of 375 mg/d EPP-AF® (GSH elevation, increased SOD activity, and reduced urinary 8-isoprostane formation). The reduction of 8-OHDG, a biomarker of DNA damage, was observed only at higher doses. Part of this phenomenon may be associated with short treatment time (7 days) and low harm potential of participants (young and healthy). There is only one study in the literature with 8-OHDG quantification in humans after propolis use. In this study, patients underwent colonoscopy, and quantification of tumor markers for colon cancer was performed on biopsy samples in two groups previously receiving placebo or propolis. There was no benefit from propolis use for tumor markers, and there was an increase in 8-OHDG concentration in patients receiving propolis [42]. This effect may have been caused by the preparation of colonoscopy, which used laxative agents that may have promoted cell damage. The biochemical tests, done 30 days after the last dose of 750 mg/d of EPP-AF®, did not show any hematologic, metabolic changes or either kidney or hepatic damage compared with baseline. In addition, the frequency of adverse events was low (3 cases, 8.1%), all mild and potentially unrelated to EPP-AF® use. In this context, the present work shows that EPP-AF® at doses up to 750 mg/d is safe and does not promote oxidative or toxic effects, at least in healthy humans.

The main limitations of this study are in its design. As it was a “proof of concept” study, a “before and after” design was chosen. There were no blinding, randomization, or placebo control, which may inflate the magnitude of the observed effect. Also, doses were elevated without a wash-out period and a cumulative dose effect may have occurred. The safety concerns of EPP-AF® use could not be generalized, since we only studied a small group of young humans, without underlying diseases or concomitant use of drugs. Despite these limitations, the study's strengths are to use healthy young people at different dose exposures, with a set of biomarkers being considered as the gold standard for detecting oxidative stress injury in humans and performed with a standardized presentation form of propolis.

In conclusion, the present study shows, for the first time, that a standardized propolis extract led to a reduction in urinary excretion of 8-isoprostanes, which has been correlated with a rate of lipid peroxidation, and reduction in 8-OHDG, which has been shown to be a reliable marker of oxidative DNA damage in humans, associated with increased enzymatic protection capacity for redox damage.

## Figures and Tables

**Table 1:** Biochemical safety profile before (baseline) and 30 days after the end of EPP-AF® (750 mg/d). Values are expressed as mean ± SD.

	Baseline	After EPP-AF®	*P*-value
Hemoglobin (g/dL)	13.6 ± 1.6	13.5 ± 1.4	0.99
Hematocrit (%)	40.5 ± 4.4	40.5 ± 4.0	0.41
Creatinine (mg/dL)	0.89 ± 0.14	0.87 ± 0.14	0.06
Urea (mg/dL)	26.4 ± 7.4	27.5 ± 6.8	0.28
Direct bilirrubin (mg/dL)	0.16 ± 0.06	0.17 ± 0.07	0.06
Aspartate aminotransferase (AST, U/L)	17.4 ± 3.8	17.6 ± 3.8	0.82
Alcaline phosphatase (U/L)	142.7 ± 45.8	148.7 ± 49.0	0.08
Gamma glutamyl-transferase (U/L)	24 ± 14	25 ± 13.6	0.20
Glycemia (mg/dL)	80.6 ± 6.4	79.4 ± 7.4	0.32

**Table 2:** FRAP (mmol/L), vitamin E (*μ*mol/L), GSH, GSSG, GSH/GSSG ratio, and SOD in plasma before (baseline) and after oral use of EPP-AF® (375 and 750 mg/d) in healthy volunteers. Values are expressed as median and interquartile 25–75% range.

	Baseline	EPP-AF 375 mg	*P*	EPP-AF 750 mg	*P*
FRAP (mmol/L)	0.67 (0.61–0.83)	0.68 (0.62–0.81)	0.07	0.69 (0.63–0.87)	0.66
Vitamin E (*μ*mol/L)	21.0 (18.3–25.3)	20.3 (16.7–24.5)	0.06	20.0 (17.1–23.8)	0.55
GSH (mmol/L)	1.23 (1.06–1.34)	1.33 (1.06–1.47)	**0.04**	1.09 (1.02–1.17)	0.29
GSSG (mmol/L)	1.89 (1.3–2.32)	2.43 (2.71–3.39)	0.45	2.02 (1.45–2.78)	0.99
GSH/GSSG	0.63 (0.57–0.86)	0.56 (0.39–0.71)	0.99	0.50 (0.41–0.76)	0.23
SOD (% inhibition)	68.8 (66.1–73.3)	78.2 (72.2–80.5)	**<0.0001**	77.7 (74.1–82.6)	**<0.0001**

*P* value compared with baseline.

**Table 3:** Urinary excretion of 8-isoprostanes (ng/mg de creatinine) and 8-OHDG (ng/mg de creatinine) and MDA plasma concentration (*μ*mol/L) before (baseline) and after oral use of EPP-AF® (375 and 750 mg/d) in health volunteers. Values are expressed as mean and confidence interval of 95% (CI 95%).

	Baseline	EPP-AF 375 mg	*P*	EPP-AF 750 mg	*P*
8-ISO (ng/mg de creatinine)	1.1 (0.9–1.3)	0.85 (0.75–0.95)	**0.01**	0.89 (0.74–1.0)	**0.03**
8-OHDG (ng/mg de creatinine)	15.7 (13.2–18.1)	13.9 (12.2–15.6)	0.16	11.6 (10.2–13.0)	**0.004**
MDA (*μ*mol/L)	2.9 (2.3–3.4)	2.6 (2.2–2.9)	0.22	3.3 (2.8–3.9)	0.06

*P* value compared with baseline.

## Data Availability

All the data supporting the conclusions of this study are included as supplementary material.

## Abbreviations

EPP-AF®: Standardized propolis extract Apis Flora
GSH: Reduced glutathione
GSSH: Oxidized glutathione
SOD: Superoxide dismutase
FRAP: Ferric reducing antioxidant power
MDA: Malondialdehyde
8-OHDG: 8-hidroxi-deoxiguanosine
8-ISO: 8-isoprostanes.

## Supplementary Materials

Supplementary Materials Table 1: GSH, GSSG (mmol/L), GSH/GSSG ratio, SOD (% inhibition), and Vitamin E (*μ*mol/L) values obtained on baseline period. Table 2: GSH, GSSG (mmol/L), GSH/GSSG ratio, SOD (% inhibition), and Vitamin E (*μ*mol/L) obtained after oral use of 375 mg/d EPP-AF® during 7±2 days. Table 3: GSH, GSSG (mmol/L), GSH/GSSG ratio, SOD (% inhibition), and Vitamin E (*μ*mol/L) obtained after oral use of 750 mg/d EPP-AF® during 7±2 days. Table 4: plasma MDA mmol/L, FRAP (mmol), 8-isoprostanes, and 8-OHDG in urine (ng/mg creatinine) obtained on baseline period. Table 5: plasma MDA (mmol/L), FRAP (mmol), 8-isoprostanes, and 8-OHDG in urine (ng/mg creatinine) obtained after oral use of 375 mg/d EPP-AF during 7±2 days. Table 6: plasma MDA (mmol/L), FRAP mmol), 8-isoprostanes, and 8-OHDG in urine (ng/mg creatinine) obtained after oral use of 375 mg/d EPP-AF during 7±2 days.
