# Supplementary material for: Antioxidant Effect of Standardized Extract of Propolis (EPP-AF®) in Healthy Volunteers: A “Before and After” Clinical Study
Source: Evid Based Complement Alternat Med. 2020 Oct 16;2020:7538232. doi: 10.1155/2020/7538232 (PMC7585652; doi:10.1155/2020/7538232)
Supplement: Supplementary Materials — Table 1: GSH, GSSG (mmol/L), GSH/GSSG ratio, SOD (% inhibition), and Vitamin E (μmol/L) values obtained on baseline period. Table 2: GSH, GSSG (mmol/L), GSH/GSSG ratio, SOD (% inhibition), and Vitamin E (μmol/L) obtained after oral use of 375 mg/d EPP-AF® during 7±2 days. Table 3: GSH, GSSG (mmol/L), GSH/GSSG ratio, SOD (% inhibition), and Vitamin E (μmol/L) obtained after oral use of 750 mg/d EPP-AF® during 7±2 days. Table 4: plasma MDA mmol/L, FRAP (mmol), 8-isoprostanes, and 8-OHDG in urine (ng/mg creatinine) obtained on baseline period. Table 5: plasma MDA (mmol/L), FRAP (mmol), 8-isoprostanes, and 8-OHDG in urine (ng/mg creatinine) obtained after oral use of 375 mg/d EPP-AF during 7±2 days. Table 6: plasma MDA (mmol/L), FRAP mmol), 8-isoprostanes, and 8-OHDG in urine (ng/mg creatinine) obtained after oral use of 375 mg/d EPP-AF during 7±2 days. [file 7538232.f1.docx]

Supplementary Material

Table 1 –GSH, GSSG (μmol/L), GSH/GSSG ratio, SOD (% inhibition) and Vitamin E (µmol/L) values obtained on baseline period.

| ID | GSH | GSSG | GSH/GSSG | SOD | Vit. E |
| --- | --- | --- | --- | --- | --- |
| 1 | 1.30 | 3.44 | 0.37 | 75.10 | 18.23 |
| 2 | 1.34 | 3.25 | 0.41 | 76.67 | 19.92 |
| 3 | 1.26 | 3.24 | 0.38 | 60.56 | 10.40 |
| 4 | 1.43 | 3.44 | 0.41 | 73.20 | 17.03 |
| 5 | 1.39 | 2.09 | 0.66 | 79.44 | 27.17 |
| 6 | 1.37 | 2.04 | 0.66 | 77.37 | 21.70 |
| 7 | 1.28 | 1.99 | 0.63 | 62.83 | 31.59 |
| 8 | 1.28 | 2.06 | 0.62 | 72.75 | 25.71 |
| 9 | 1.22 | 2.13 | 0.57 | 64.41 | 22.08 |
| 10 | 1.32 | 2.37 | 0.55 | 68.47 | 34.92 |
| 11 | 1.36 | 1.75 | 0.77 | 74.28 | 17.68 |
| 12 | 1.37 | 2.32 | 0.59 | 68.20 | 23.66 |
| 13 | 1.43 | 2.38 | 0.60 | 67.08 | 20.20 |
| 14 | 1.37 | 2.33 | 0.58 | 69.83 | 12.65 |
| 15 | 1.34 | 2.26 | 0.59 | 74.98 | 18.62 |
| 16 | 1.31 | 2.31 | 0.56 | 66.57 | 20.94 |
| 17 | 1.35 | 2.43 | 0.55 | 62.71 | 17.91 |
| 18 | 1.14 | 0.83 | 1.37 | 70.93 | 28.84 |
| 19 | 1.13 | 0.92 | 1.22 | 66.14 | 22.52 |
| 20 | 1.02 | 0.91 | 1.12 | 65.94 | 39.76 |
| 21 | 1.05 | 1.07 | 0.98 | 73.56 | 19.57 |
| 22 | 1.10 | 1.16 | 0.94 | 70.68 | 18.27 |
| 23 | 1.03 | 1.18 | 0.87 | 49.50 | 27.39 |
| 24 | 1.14 | 1.06 | 1.06 | 72.50 | 26.21 |
| 25 | 1.13 | 1.27 | 0.88 | 66.41 | 20.99 |
| 26 | 1.12 | 1.31 | 0.85 | 73.01 | 16.41 |
| 27 | 1.04 | 1.61 | 0.64 | 67.90 | 25.18 |
| 28 | 1.03 | 1.63 | 0.63 | 58.47 | 20.50 |
| 29 | 1.10 | 1.66 | 0.65 | 68.15 | 20.67 |
| 30 | 1.03 | 1.76 | 0.58 | 67.03 | 24.47 |
| 31 | 1.04 | 1.88 | 0.55 | 92.61 | 19.84 |
| 32 | 1.06 | 1.90 | 0.55 | 69.18 | 17.12 |
| 33 | 1.04 | 1.82 | 0.57 | 61.34 | 21.75 |
| 34 | 1.24 | 1.83 | 0.67 | 71.47 | 23.62 |

Table 2 – GSH, GSSG (μmol/L), GSH/GSSG ratio, SOD (% inhibition) and Vitamin E (µmol/L) obtained after orally use of 375 mg/d EPP-AF® during 7±2 days.

| ID | GSH | GSSG | GSH/GSSG | SOD | Vit. E |
| --- | --- | --- | --- | --- | --- |
| 1 | 1.35 | 2.71 | 0.49 | 70.78 | 12.62 |
| 2 | 1.47 | 2.49 | 0.59 | 80.38 | 19.93 |
| 3 | 1.52 | 2.37 | 0.64 | 82.03 | 9.27 |
| 4 | 1.66 | 2.08 | 0.79 | 81.23 | 16.74 |
| 5 | 1.55 | 2.94 | 0.52 | 79.31 | 23.71 |
| 6 | 1.46 | 2.03 | 0.72 | 80.47 | 24.27 |
| 7 | 1.45 | 2.09 | 0.69 | 67.61 | 21.59 |
| 8 | 1.48 | 2.16 | 0.68 | 80.16 | 25.11 |
| 9 | 1.52 | 2.14 | 0.71 | 78.55 | 21.00 |
| 10 | 1.52 | 2.09 | 0.72 | 77.64 | 32.58 |
| 11 | 1.67 | 2.07 | 0.80 | 78.09 | 18.75 |
| 12 | 1.00 | 1.59 | 0.62 | 78.69 | 26.66 |
| 13 | 0.94 | 1.71 | 0.55 | 56.88 | 22.46 |
| 14 | 0.96 | 1.70 | 0.56 | 70.36 | 11.80 |
| 15 | 0.97 | 1.39 | 0.69 | 83.16 | 20.13 |
| 16 | 0.89 | 1.44 | 0.61 | 78.57 | 19.40 |
| 17 | 1.03 | 1.70 | 0.60 | 64.70 | 18.98 |
| 18 | 1.32 | 3.33 | 0.39 | 85.93 | 27.46 |
| 19 | 1.32 | 3.46 | 0.38 | 74.95 | 21.17 |
| 20 | 1.27 | 3.29 | 0.38 | 68.32 | 37.31 |
| 21 | 1.29 | 3.34 | 0.38 | 82.36 | 18.87 |
| 22 | 1.34 | 3.27 | 0.41 | 72.69 | 20.67 |
| 23 | 1.43 | 3.13 | 0.45 | 79.23 | 27.07 |
| 24 | 1.40 | 3.92 | 0.35 | 73.27 | 23.61 |
| 25 | 1.30 | 3.63 | 0.35 | 68.66 | 16.53 |
| 26 | 1.22 | 3.45 | 0.35 | 80.58 | 13.80 |
| 27 | 1.34 | 3.51 | 0.38 | 76.38 | 25.51 |
| 28 | 1.30 | 3.52 | 0.36 | 74.12 | 14.96 |
| 29 | 1.31 | 3.37 | 0.38 | 82.88 | 18.33 |
| 30 | 1.38 | 3.58 | 0.38 | 78.35 | 20.49 |
| 31 | 1.50 | 3.80 | 0.39 | 93.18 | 17.66 |
| 32 | 1.05 | 0.92 | 1.14 | 74.80 | 15.55 |
| 33 | 1.06 | 0.93 | 1.13 | 64.50 | 15.93 |
| 34 | 1.05 | 1.01 | 1.03 | 76.62 | 25.93 |

Table 3 –GSH, GSSG (μmol/L), GSH/GSSG ratio, SOD (% inhibition) and Vitamin E (µmol/L) obtained after orally use of 750 mg/d EPP-AF® during 7±2 days.

| ID | GSH | GSSG | GSH/GSSG | SOD | Vit. E |
| --- | --- | --- | --- | --- | --- |
| 1 | 0.94 | 1.49 | 0.63 | 66.40 | 15.18 |
| 2 | 1.12 | 1.68 | 0.66 | 79.62 | 21.35 |
| 3 | 1.01 | 2.35 | 0.43 | 70.96 | 10.21 |
| 4 | 1.04 | 2.13 | 0.48 | 63.72 | 18.29 |
| 5 | 1.02 | 2.05 | 0.49 | 83.36 | 19.55 |
| 6 | 0.95 | 0.88 | 1.08 | 78.26 | 22.54 |
| 7 | 1.02 | 2.03 | 0.50 | 73.10 | 17.35 |
| 8 | 0.95 | 2.02 | 0.47 | 77.89 | 23.72 |
| 9 | 0.95 | 2.27 | 0.41 | 75.72 | 22.66 |
| 10 | 1.00 | 2.23 | 0.44 | 78.55 | 31.03 |
| 11 | 1.25 | 2.68 | 0.46 | 84.12 | 20.31 |
| 12 | 1.18 | 2.63 | 0.44 | 77.13 | 32.28 |
| 13 | 1.12 | 2.95 | 0.37 | 67.10 | 23.51 |
| 14 | 1.14 | 2.78 | 0.40 | 86.21 | 13.70 |
| 15 | 1.18 | 2.79 | 0.42 | 80.82 | 19.42 |
| 16 | 1.15 | 3.28 | 0.34 | 82.37 | 20.62 |
| 17 | 1.15 | 3.03 | 0.37 | 76.25 | 15.61 |
| 18 | 0.99 | 0.87 | 1.13 | 80.46 | 32.13 |
| 19 | 1.06 | 0.87 | 1.21 | 74.99 | 19.50 |
| 20 | 1.05 | 0.85 | 1.23 | 83.51 | 42.45 |
| 21 | 1.09 | 0.99 | 1.10 | 80.44 | 17.28 |
| 22 | 1.09 | 1.02 | 1.06 | 74.49 | 17.20 |
| 23 | 1.17 | 1.46 | 0.79 | 84.87 | 24.42 |
| 24 | 1.07 | 1.42 | 0.75 | 75.77 | 24.05 |
| 25 | 1.05 | 1.47 | 0.71 | 80.49 | 16.13 |
| 26 | 1.09 | 1.55 | 0.70 | 77.53 | 16.85 |
| 27 | 1.08 | 1.77 | 0.61 | 77.53 | 28.17 |
| 28 | 1.03 | 1.61 | 0.63 | 62.27 | 21.30 |
| 29 | 1.11 | 1.52 | 0.73 | 89.68 | 19.79 |
| 30 | 1.19 | 1.36 | 0.87 | 84.14 | 25.89 |
| 31 | 1.46 | 3.70 | 0.39 | 90.88 | 16.87 |
| 32 | 1.34 | 3.78 | 0.35 | 63.98 | 14.50 |
| 33 | 1.33 | 3.79 | 0.35 | 66.15 | 18.76 |
| 34 | 1.25 | 3.94 | 0.31 | 76.41 | 23.48 |

Table 4 – Plasma MDA μmol/L, FRAP (mmol), 8-isoprostanes and 8-OHDG in urine (ng/mg creatinine) obtained on baseline period.

| ID | MDA | FRAP | 8-ISO | 8-OHDG |
| --- | --- | --- | --- | --- |
| 1 | 6.91 | 0.55 | 0.80 | 3.94 |
| 2 | 3.80 | 0.86 | 0.52 | 10.26 |
| 3 | 4.10 | 0.70 | 0.99 | 16.23 |
| 4 | 3.41 | 0.93 | 1.24 | 9.14 |
| 5 | 3.75 | 0.90 | 0.75 | 8.11 |
| 6 | 5.92 | 0.60 | 0.70 | 32.67 |
| 7 | 4.12 | 0.83 | 0.68 | 5.12 |
| 8 | 3.25 | 0.74 | 0.69 | 18.20 |
| 9 | 3.93 | 0.46 | 1.78 | 27.76 |
| 10 | 3.71 | 0.98 | 0.85 | 15.84 |
| 11 | 5.27 | 0.89 | 1.37 | 20.32 |
| 12 | 4.01 | 0.93 | 1.20 | 12.79 |
| 13 | 4.08 | 0.65 | 0.70 | 10.59 |
| 14 | 3.90 | 0.85 | 0.78 | 10.28 |
| 15 | 2.80 | 0.57 | 1.13 | 18.37 |
| 16 | 3.56 | 0.51 | 0.81 | 12.40 |
| 17 | 3.61 | 0.57 | 1.40 | 13.85 |
| 18 | 1.24 | 0.67 | 1.10 | 17.86 |
| 19 | 1.42 | 0.78 | 0.79 | 7.98 |
| 20 | 1.46 | 1.16 | 1.88 | 23.49 |
| 21 | 0.79 | 0.70 | 0.67 | 16.28 |
| 22 | 1.76 | 0.67 | 2.56 | 15.74 |
| 23 | 2.26 | 0.76 | 0.79 | 15.69 |
| 24 | 0.84 | 0.67 | 0.78 | 7.91 |
| 25 | 1.61 | 0.60 | 0.83 | 8.66 |
| 26 | 0.89 | 0.64 | 0.81 | 19.03 |
| 27 | 2.58 | 0.77 | 1.25 | 13.01 |
| 28 | 1.34 | 0.58 | 0.89 | 20.87 |
| 29 | 0.96 | 0.72 | 1.17 | 17.89 |
| 30 | 2.61 | 1.00 | 3.31 | 28.56 |
| 31 | 2.76 | 1.11 | 0.99 | 19.59 |
| 32 | 2.14 | 0.88 | 1.93 | 30.42 |
| 33 | 0.78 | 0.79 | 0.67 | 10.86 |
| 34 | 2.24 | 0.68 | 0.72 | 12.81 |

Table 5 –Plasma MDA (μmol/L), FRAP (mmol), 8-isoprostanes and 8-OHDG in urine (ng/mg creatinine) obtained after orally use of 375 mg/d EPP-AF during 7±2 days.

| ID | MDA | FRAP | 8-ISO | 8-OHDG |
| --- | --- | --- | --- | --- |
| 1 | 3.65 | 0.57 | 0.83 | 4.37 |
| 2 | 3.59 | 0.81 | 0.65 | 9.48 |
| 3 | 2.58 | 0.67 | 1.05 | 24.70 |
| 4 | 3.54 | 0.90 | 0.84 | 11.31 |
| 5 | 3.98 | 0.85 | 1.11 | 8.18 |
| 6 | 3.06 | 0.58 | 0.78 | 17.19 |
| 7 | 4.67 | 0.76 | 0.72 | 11.96 |
| 8 | 4.91 | 0.66 | 0.51 | 20.13 |
| 9 | 3.29 | 0.44 | 0.83 | 15.31 |
| 10 | 4.51 | 1.00 | 0.78 | 15.37 |
| 11 | 3.35 | 0.75 | 1.34 | 10.08 |
| 12 | 3.83 | 0.79 | 0.92 | 11.16 |
| 13 | 3.03 | 0.72 | 0.45 | 7.09 |
| 14 | 2.83 | 0.94 | 0.53 | 6.20 |
| 15 | 1.88 | 0.62 | 0.81 | 15.95 |
| 16 | 1.69 | 0.52 | 0.71 | 16.71 |
| 17 | 1.82 | 0.70 | 1.10 | 12.86 |
| 18 | 2.65 | 0.63 | 0.60 | 13.18 |
| 19 | 1.85 | 0.70 | 0.50 | 17.52 |
| 20 | 1.50 | 1.21 | 0.80 | 15.31 |
| 21 | 1.61 | 0.63 | 1.25 | 11.62 |
| 22 | 0.76 | 0.66 | 1.69 | 18.23 |
| 23 | 2.02 | 0.65 | 0.80 | 18.42 |
| 24 | 2.27 | 0.43 | 1.20 | 14.18 |
| 25 | 2.49 | 0.67 | 0.56 | 16.47 |
| 26 | 1.51 | 0.61 | 0.75 | 15.72 |
| 27 | 1.57 | 0.69 | 1.09 | 23.23 |
| 28 | 1.65 | 0.60 | 0.64 | 21.80 |
| 29 | 2.20 | 0.87 | 0.77 | 12.85 |
| 30 | 1.19 | 0.82 | 1.36 | 15.09 |
| 31 | 2.53 | 1.05 | 0.58 | 6.82 |
| 32 | 1.82 | 0.72 | 0.74 | 14.64 |
| 33 | 1.61 | 0.59 | 0.74 | 6.38 |
| 34 | 1.24 | 0.64 | 0.91 | 14.00 |

Table 6 –Plasma MDA (μmol/L), FRAP mmol), 8-isoprostanes and 8-OHDG in urine (ng/mg creatinine) obtained after orally use of 375 mg/d EPP-AF during 7±2 days.

| ID | MDA | FRAP | 8-ISO | 8-OHDG |
| --- | --- | --- | --- | --- |
| 1 | 3.86 | 0.62 | 1.09 | 7.54 |
| 2 | 4.14 | 0.89 | 0.93 | 10.83 |
| 3 | 2.94 | 0.71 | 1.05 | 7.99 |
| 4 | 3.73 | 0.97 | 1.36 | 8.40 |
| 5 | 6.74 | 0.85 | 2.01 | 8.59 |
| 6 | 5.44 | 0.64 | 0.73 | 11.32 |
| 7 | 4.35 | 0.74 | 0.90 | 7.59 |
| 8 | 5.44 | 0.76 | 0.47 | 11.39 |
| 9 | 5.01 | 0.52 | 0.66 | 10.77 |
| 10 | 5.01 | 1.08 | 0.87 | 9.40 |
| 11 | 4.84 | 0.96 | 1.29 | 12.86 |
| 12 | 5.83 | 0.96 | 0.91 | 8.20 |
| 13 | 5.22 | 0.70 | 0.49 | 9.17 |
| 14 | 4.69 | 0.88 | 0.59 | 6.25 |
| 15 | 3.85 | 0.66 | 0.90 | 11.37 |
| 16 | 3.41 | 0.56 | 0.71 | 6.77 |
| 17 | 3.64 | 0.62 | 0.45 | 11.14 |
| 18 | 2.57 | 0.67 | 0.82 | 12.17 |
| 19 | 2.73 | 0.64 | 0.61 | 24.62 |
| 20 | 1.88 | 1.05 | 0.75 | 8.65 |
| 21 | 1.54 | 0.64 | 0.26 | 10.36 |
| 22 | 2.74 | 0.53 | 1.67 | 20.79 |
| 23 | 4.26 | 0.64 | 0.48 | 11.82 |
| 24 | 1.63 | 0.54 | 0.43 | 13.90 |
| 25 | 2.73 | 0.64 | 0.99 | 12.41 |
| 26 | 1.95 | 0.60 | 0.79 | 12.34 |
| 27 | 1.18 | 0.61 | 0.90 | 11.09 |
| 28 | 1.94 | 0.00 | 1.05 | 13.88 |
| 29 | 1.86 | 0.90 | 0.76 | 15.00 |
| 30 | 0.99 | 0.85 | 2.31 | 17.64 |
| 31 | 1.93 | 0.98 | 0.31 | 8.00 |
| 32 | 1.76 | 0.82 | 1.13 | 18.58 |
| 33 | 1.53 | 0.60 | 1.01 | 12.44 |
| 34 | 1.81 | 0.59 | 0.73 | 10.93 |
